# Preventing Parastomal Hernias After Radical Cystectomy with Ileal Conduit: A Systematic Review Regarding Surgical Prophylactic Techniques

**DOI:** 10.3390/jpm16010040

**Published:** 2026-01-08

**Authors:** Giulio Rossin, Arianna Biasatti, Ioana Alexandra Iachimovsky, Luca Braulin, Alessandro Zucchi, Tommaso Cai, Antonio Vitarelli, Michele Rizzo, Paolo Umari, Giovanni Liguori

**Affiliations:** 1 Urological Clinic, Department of Medical, Surgical and Health Sciences, Cattinara Hospital, University of Trieste, 34127 Trieste, Italy; giulio.rossin@asugi.sanita.fvg.it (G.R.); arianna.biasatti@asugi.sanita.fvg.it (A.B.); ioana.iachimovsky@asugi.sanita.fvg.it (I.A.I.); luca.braulin@asugi.sanita.fvg.it (L.B.); michele.rizzo@asugi.sanita.fvg.it (M.R.); paolo.umari@asugi.sanita.fvg.it (P.U.); 2 Department of Urology, University of Pisa, 56124 Pisa, Italy; alessandro.zucchi@unipi.it; 3 Department of Urology, Santa Chiara Hospital, 38122 Trento, Italy; tommaso.cai@unitn.it; 4 Department of Urology, Mater Dei Hospital, 70125 Bari, Italy; antonio.vitarelli@hotmail.com

**Keywords:** parastomal hernia, radical cystectomy, prevention, mesh

## Abstract

**Background/Objective**: Parastomal hernia (PSH) following radical cystectomy (RC) with ileal conduit represents a significant late complication. Preventive strategies have been described but are not yet routinely incorporated into clinical practice. We conducted a systematic review of the current literature to assess the efficacy of PSH preventive techniques for ileal conduit. **Methods**: A literature search of PubMed/MEDLINE, Scopus, CENTRAL, and Web of Science databases was conducted from 2010 to December 2024 following PRISMA guidelines. Inclusion criteria were patients undergoing RC with ileal conduit, evaluation of at least one PSH preventive strategy and reporting of PSH incidence or relevant postoperative outcomes. Eligible designs included RCTs and non-randomized cohort studies. Exclusion criteria included urinary diversions other than ileal conduit, non-bladder-related indications, non-extractable outcome data, and non-original publications. **Results**: Three randomized controlled trials (RCTs) and nine non-randomized studies were included in the analysis. Studies investigating both mesh and non-mesh preventive techniques were considered. Clinical PSH recurrence rates following mesh placement ranged from 0.0% to 11.1% among the included studies. RCTs using mesh placement reported conflicting conclusions regarding its protective effects. For non-mesh preventive strategies, clinical PSH recurrence rates ranged from 0.0% to 11.5%. The only RCT focusing on non-mesh approaches reported positive protective effects for the experimental group. All procedures were safe, with no significant increase in complication rates compared to conventional interventions. **Conclusions**: The low quality of current evidence prevents definitive conclusions regarding the protective effects of both mesh and non-mesh preventive approaches. High-quality evidence is needed to make conclusive statements on this topic. Patients at high risk for PSH development should be offered personalized preoperative counselling and the opportunity to participate in ongoing RCTs.

## 1. Introduction

Radical cystectomy (RC) remains the gold-standard treatment for muscle-invasive bladder cancer (MIBC) [1]. Despite the introduction of minimally invasive techniques, including robot-assisted approaches, RC continues to be associated with substantial postoperative morbidity [2,3]. Among the long-term complications following RC with ileal conduit urinary diversion, parastomal hernia (PSH) is one of the most clinically relevant. PSH is defined as the protrusion of intra-abdominal contents through the abdominal wall adjacent to the stoma and represents the most frequently reported stomal complication after RC [4,5], with incidence rates ranging from 17% to more than 30% [6,7,8,9]. Although many patients with PSH remain asymptomatic [4], the condition can markedly affect quality of life due to associated symptoms and functional limitations. The most commonly reported indications for re-intervention after RC include abdominal discomfort, abdominal pain, and difficulty maintaining adequate ostomy appliance fit [10,11,12]. In less frequent cases, PSH may lead to severe complications such as fistula formation, stomal stenosis, acute strangulation, or complete small bowel obstruction [11].

Despite these considerations, the absence of consistent evidence supporting the benefits of prophylactic interventions has limited their widespread adoption. Moreover, previous systematic reviews have not explored this topic comprehensively, restricting the application of these techniques largely to experimental settings. Nonetheless, interest in preventive surgical strategies has increased in recent years. In the context of personalized medicine, prophylactic mesh placement has been explored as a strategy to reduce postoperative PSH incidence [13], and several innovative surgical approaches have recently been described with the same objective. The aim of this systematic review is therefore to synthesize the most current evidence regarding prophylactic interventions performed during RC with ileal conduit urinary diversion.

## 2. Materials and Methods

### 2.1. Literature Search

A systematic review was conducted in accordance with the Preferred Reporting Items for Systematic Reviews and Meta-Analyses (PRISMA) guidelines [14]. The PRISMA checklist is provided in the Supplementary Materials as File S1. The review protocol was registered in the International Prospective Register of Systematic Reviews (PROSPERO) database (CRD42024595747). A comprehensive bibliographic search was carried out in December 2024 across the PubMed/Medline, Scopus, Cochrane Central Register of Controlled Trials (CENTRAL), and Web of Science databases to identify relevant manuscripts. The detailed search strategy is provided in the Supplementary Materials File S2. The research question was formulated using the PICO framework. The Population comprised patients undergoing RC with ileal conduit urinary diversion; the Intervention consisted of prophylactic surgical techniques intended to prevent the development of PSH; the Comparator was conventional RC performed without such preventive measures; and the Outcomes included the incidence of PSH during follow-up, in addition to perioperative outcomes and complication rates associated with the prophylactic procedures. No research filters were applied to ensure that no potentially valuable information was excluded.

### 2.2. Study Selection

Two reviewers (G.R. and I.A.I.) independently assessed all studies according to the predetermined inclusion and exclusion criteria derived from the PICO framework.

Studies were included if they involved patients undergoing RC with ileal conduit urinary diversion performed via open, laparoscopic, or robotic approaches. Eligible studies assessed at least one PSH prevention strategy, encompassing both mesh and non-mesh techniques. Studies were required to report the incidence of PSH, defined clinically or radiologically, or to provide data for any relevant secondary outcome. Eligible study designs consisted of randomized controlled trials (RCTs) and non-randomized investigations, including prospective and retrospective cohort studies.

Studies were excluded if they involved urinary diversions other than ileal conduit (e.g., continent diversions or cutaneous ureterostomy) or if they examined general surgery interventions for non-bladder-related diseases (e.g., colorectal conditions). Studies lacking extractable outcome data were deemed ineligible. Case reports, systematic reviews, meta-analyses, expert opinions, letters to the editor, conference communications, conference abstracts, and editorials were also excluded.

### 2.3. Outcome Measurement

**Figure 1:**
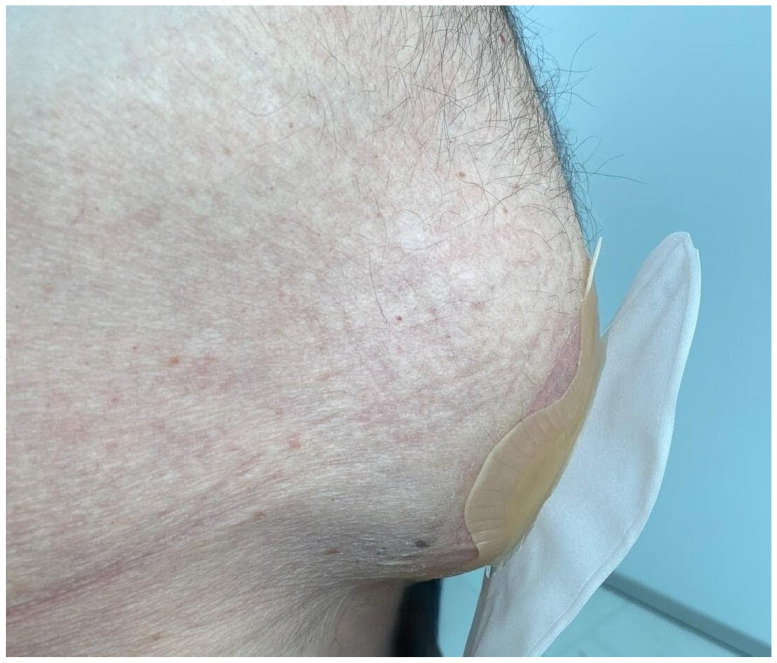
Clinical presentation of parastomal hernia at the ileal conduit stoma site.

**Figure 2:**
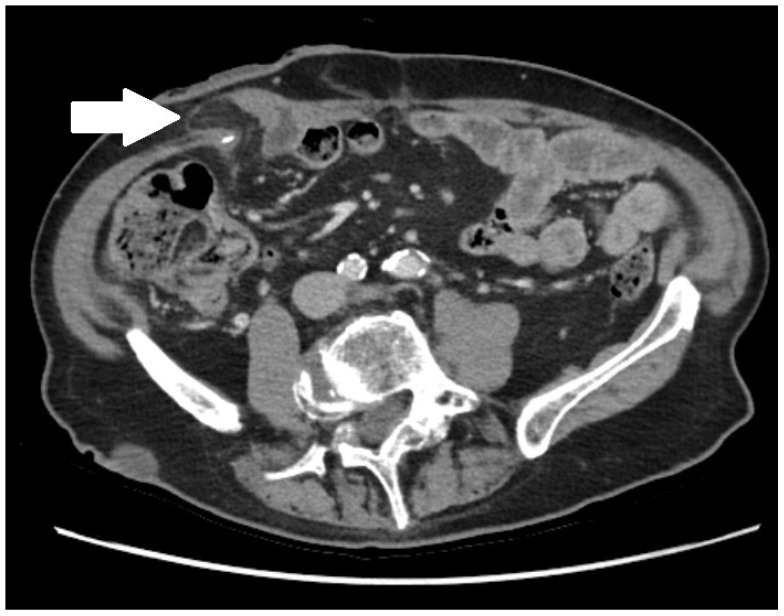
Radiological presentation of parastomal hernia at the ileal conduit stoma site (the arrow points towards a Type 0 parastomal hernia according to the Moreno-Matias classification).

The primary outcome was the recurrence rate of PSH following the index procedure. PSH was defined on the basis of clinical and/or radiological assessment at follow-up. Clinically, PSH was identified as a palpable bulge at the stoma site associated with a fascial defect (Figure 1). Radiologically, PSH was classified according to the Moreno-Matias system using computed tomography (CT) imaging [15] (Figure 2). Secondary outcomes included length of hospital stay (days), 90-day morbidity (assessed using the Clavien–Dindo classification), mesh-related complication rates, and the rate of re-operation. Postoperative morbidity was defined as any deviation from the expected postoperative course, whereas severe complications were defined as Clavien–Dindo grade ≥ III events.

### 2.4. Data Extraction

The systematic search was independently conducted by two reviewers (G.R., I.A.I.) following a standardized procedure. Discrepancies during the title and abstract screening stages were resolved by inclusion of the study. Discrepancies during full-text evaluation were resolved through discussion between the two reviewers; if consensus could not be reached, a third reviewer (G.L.) was consulted. Data extraction was performed independently by the same reviewers using a dedicated data-collection template. The extraction sheet was developed in Microsoft Excel (Microsoft, Redmond, WA, USA). Retrieved data included: study characteristics (author, year of publication, study design), patient demographics (age, BMI), treatment details (operative approach, index procedure, surgical technique), perioperative outcomes (general and procedure-specific complications, length of stay), and follow-up parameters (duration, radiological and clinical recurrence rates, and re-operation rates). When required, study authors were contacted to clarify missing or ambiguous data. A pooled or meta-analytic synthesis was not feasible due to the heterogeneity of study designs and reported outcomes.

### 2.5. Study Quality

Methodological quality was assessed independently by two reviewers (G.R., I.A.I.). The Critical Appraisal Skills Programme (CASP) Randomized Controlled Trials Checklist was used to evaluate RCTs [16]. Non-randomized studies were assessed using the Methodological Index for Non-Randomized Studies (MINORS) tool [17]. Both reviewers independently evaluated the included manuscripts. Discrepancies were resolved through discussion; if disagreement persisted, a third senior reviewer (G.L.) was consulted.

## 3. Results

**Figure 3:**
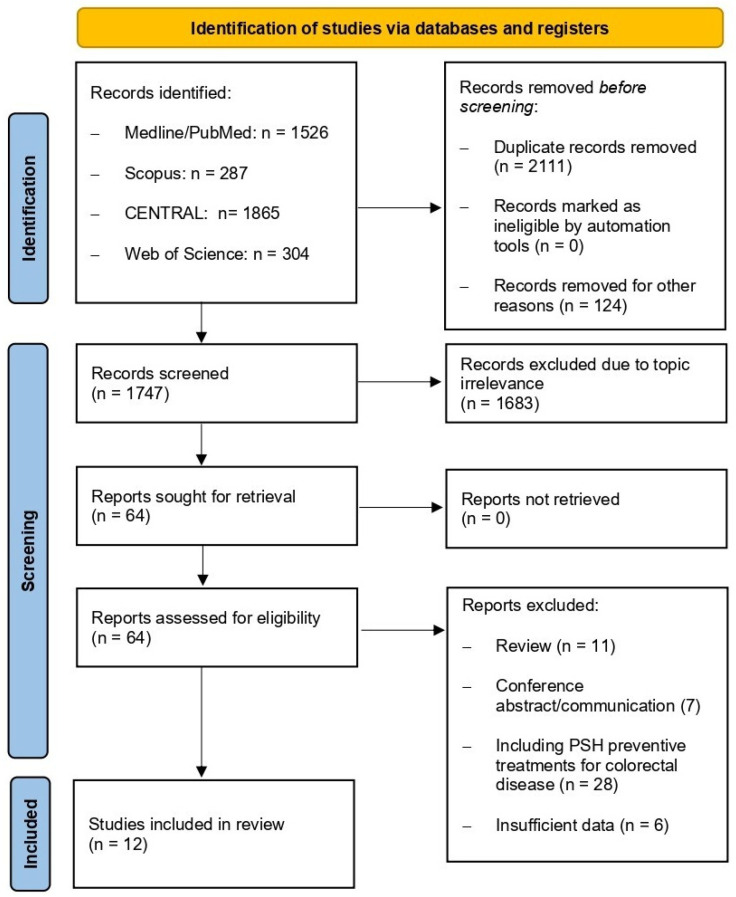
PRISMA flow diagram of the included studies.

The study selection process is summarized in the PRISMA flow diagram (Figure 3). A total of 12 studies met the inclusion criteria and were incorporated into the qualitative analysis. The included manuscripts comprised 3 RCTs [13,18,19] and 9 non-randomized studies [20,21,22,23,24,25,26,27,28]. An overview of the main characteristics of these studies is provided in Table 1.

### 3.1. Parastomal Hernia Prevention with Prophylactic Mesh Placement

The main findings regarding PSH prevention with prophylactic mesh placement are summarized in Table 2. All included studies described mesh placement in the sublay position using the Keyhole technique. Overall postoperative complications and mesh-related complications were reported separately. Two RCTs addressed this topic. The first, conducted by Liedberg et al. [13], compared 118 patients undergoing RC with ileal conduit and prophylactic mesh placement with a control cohort receiving conventional RC with ileal conduit. All procedures were performed via open surgery. At a median follow-up of 24 months, the mesh cohort reported one mesh-related complication (0.8%) and a clinical PSH recurrence rate of 11.0%, compared with 23.0% in the control arm [13]. More recently, Djaladat et al. published an RCT including 72 patients undergoing RC with prophylactic biologic mesh placement [18]. At a median follow-up of 24 months, eight patients in each group developed clinical PSH. Radiological recurrence occurred in 19 patients in the control group and 18 patients in the mesh group, corresponding to PSH-free survival rates of 74% vs. 75% at 1 year and 69% vs. 62% at 2 years for the mesh and control cohorts, respectively. Median operative time was 332 min in the control group and 362 min in the mesh group, while median length of stay was 5 days in both groups. No mesh-related complications were reported. Styrke et al. described a series of 114 patients treated with RC plus prophylactic mesh placement [24], reporting a recurrence rate of 7% at a median follow-up of 32 months; no high-grade complications or mesh-related events were observed. Jiang et al. reported 34 robot-assisted procedures among 38 total cases [22]. No severe postoperative complications occurred, and the recurrence rate was 2.6% at a 4-month follow-up.

### 3.2. Parastomal Hernia Prevention Without Prophylactic Mesh Placement

The main findings regarding PSH prevention without prophylactic mesh placement are summarized in Table 3. The primary strategies investigated were extraperitonealization (EP) of the ileal conduit [20,27], and a novel technique for ileal conduit creation [25]. Zhou et al. recently published an RCT evaluating the EP technique [19]: At a median follow-up of 32 months, the radiological recurrence rate was 11.5% in the EP cohort versus 28.8% in the control group (HR 0.374, 95% CI 0.145–0.965). The EP approach was previously investigated by Li et al. in a retrospective cohort of 375 patients undergoing RC [26]. Radiological PSH occurred in 7.5% of patients in the EP cohort compared with 21.1% in the conventional cohort. More recently, Tanaka et al. described a novel ileal urostomy creation technique [25]. At a median follow-up of 22 months, the modified technique was associated with a 3.5% recurrence rate compared with 19.6% in the control group (HR 0.17, 95% CI 0.04–0.83).

### 3.3. Quality Assessment

The overall quality of evidence was rated as low, primarily due to the absence of prospective study designs, small sample sizes, lack of sample size calculations, unblinded outcome assessments, and significant follow-up attrition rates. A detailed assessment of potential biases is provided in the Supplementary Materials, Table S1 [13,18,19] and Table S2 [20,21,22,23,24,25,26,27,28].

## 4. Discussion

In this review, we aimed to evaluate the current state of surgical preventive strategies for ileal conduit PSH. PSH following RC with ileal conduit remains a significantly under-treated clinical issue [29]. This condition results in a general decline in quality of life for patients and increases healthcare costs for communities [29,30].

Parastomal hernia management ranges from conservative to surgical approaches depending on symptom severity. Watchful waiting is common in asymptomatic patients, although this strategy can lead to severe complications, such as small bowel strangulation or urinary stoma obstruction, and increased complexity of later surgical repair. Mild symptoms can often be addressed through specialist stoma care and lifestyle measures, including weight reduction.

Reparative surgery is indicated in the presence of significant pain, prolapse, or difficulty in maintaining an adequate appliance seal [31]. Such interventions have been extensively described in the field of general surgery, particularly following colostomy or ileostomy procedures [32,33,34]. Multiple reparative surgical approaches have been described. Early attempts involved direct suturing of the hernial defect; however, these strategies have been largely abandoned due to high recurrence rates [32]. In 1985, Sugarbaker introduced a mesh-based technique for PSH repair using a laparotomic intraperitoneal approach [35]. This technique was subsequently refined, with the mesh placed in an intraperitoneal onlay position to reduce contamination risk [36]. The Keyhole technique represents an alternative strategy, positioning the stoma through a centrally fenestrated mesh in a sublay configuration [37]. More recently, novel approaches—such as the Pauli and sandwich techniques—have been proposed, combining principles of prior methods into innovative strategies that have demonstrated encouraging preliminary results [38,39].

In patients with urostomies, PSH repair must consider the unique anatomical and surgical characteristics of the ileal conduit [33]: the presence of uretero-intestinal anastomoses and the reconfigured ileal segment increase operative complexity; moreover, the ileal loop is supported by terminal vascularization, necessitating meticulous handling during surgery. The mesentery of the ileal conduit is often short, complicating mobilization required for approaches such as the Sugarbaker technique. Additionally, RC for MIBC involves peritoneal excision below the arcuate line to achieve oncological radicality, further complicating extraperitoneal mesh placement. Consequently, PSH associated with an ileal conduit entails unique anatomical and functional considerations and should therefore be evaluated separately from PSH occurring in other stoma types. However, such differentiation is seldom undertaken, largely because ileal conduit–related PSH represents a relatively uncommon clinical scenario compared with hernias arising from other stoma sites, reflecting the substantially lower number of urostomy procedures performed relative to other stoma types. One of the few systematic reviews on this topic, conducted by Dewulf et al., underscored the considerable heterogeneity in the reported outcomes of reparative techniques for ileal conduit–associated PSH, with recurrence rates ranging from 0% to 80% [40]. The authors concluded that the optimal reparative treatment remains undefined due to considerable heterogeneity and poor evidence quality.

Given the clinical significance of PSH and the inconsistent outcomes of reparative interventions, preventive strategies are attracting increasing interest [41]: general non-operative recommendations, such as BMI reduction and preoperative optimization of immuno-nutritional status, may help mitigate PSH risk [4,42]. Among surgical strategies, prophylactic mesh placement has emerged as a promising option, while newly developed preventive techniques have likewise been introduced with the aim of mitigating PSH incidence. In this scenario, given the substantial inter-individual variability in the anatomical and functional characteristics of the ileal conduit, the adoption of personalized surgical planning—consistent with the principles of precision medicine—is warranted. Accordingly, this review aimed to synthesize the available evidence on prophylactic surgical strategies for PSH prevention, thereby supporting more individualized preoperative counselling at the time of RC, facilitating the identification of candidates most likely to benefit from such interventions, and ultimately reducing PSH risk in susceptible individuals.

### 4.1. Results on Parastomal Hernia Prevention Following Prophylactic Interventions

PSH recurrence rates following prophylactic interventions ranged from 0.0% to 18.2% across the included studies. Three RCTs have evaluated the role of prophylactic mesh in PSH prevention. Liedberg et al. reported a significantly reduced risk of clinical PSH following RC with ileal conduit in the mesh cohort compared with controls at three years (11% vs. 23%; HR 0.45, 95%CI 0.24–0.86) [13]. Saha et al. similarly reported lower PSH incidence in the mesh cohort (8.8% vs. 21.5%; *p* = 0.014) [30]; however, these results were derived from the same patient cohort and were therefore excluded to avoid duplication. In contrast, the recent RCT by Djaladat et al. [18] demonstrated no significant difference in PSH prevention using biologic mesh. PSH-free survival at one and two years was similar between the mesh and control groups (74% vs. 75% and 69% vs. 62%, respectively). Nonetheless, the authors noted potential long-term benefits of mesh reinforcement, suggesting that prophylactic mesh placement may be more appropriate for patients with longer life expectancy. Preoperative risk stratification is warranted, as prior laparotomy and elevated BMI are established independent predictors of PSH following RC with ileal conduit [7]. Accordingly, selected patients may benefit from counselling regarding prophylactic mesh placement. A recent meta-analysis found no long-term benefit of prophylactic mesh placement in preventing PSH after end colostomy [43]. Nevertheless, prophylactic mesh in ileal conduit formation may confer mild long-term protective effects, although follow-up durations in current studies remain insufficient for definitive conclusions. Conversely, Hinojosa-Gonzalez et al. demonstrated significant protective effects of mesh placement in both ostomies (OR 0.52 [0.35, 0.77], *p* = 0.001) and ileal conduit (OR 0.49 [0.25, 0.97], *p* = 0.04) subgroups [44]. However, only clinical PSH onset was considered as the outcome of interest in the ileal conduit subgroup analysis. Such heterogeneity in outcome definitions may introduce substantial bias, as noted by the authors [44], particularly given that the incidence of radiological PSH was generally higher than that of clinical PSH in the included RCTs [13,18]. Additionally, the inclusion of both Liedberg and Saha RCTs may have inflated effect estimates due to overlapping cohorts. More robust trials with long-term follow-up are required.

On the other hand, several authors have proposed innovative mesh-free surgical approaches aimed at preventing PSH formation. Recurrence rates in these sub-cohorts ranged from 0.0% to 12.2%, suggesting potential adjunctive benefits. Earlier preventive methods, such as anterior fascial fixation of the ileal conduit, yielded poor outcomes and have therefore been abandoned [45]. However, new approaches have been recently described in literature Among these, EP of the ileal conduit at the time of RC represents a promising alternative. The technique was first reported by Zhang et al. [28] and involves complete EP of the ileal conduit: a blunt dissection of the lateral peritoneal layer and abdominal wall is performed to create a tunnel for conduit placement, after which the ileal loop is secured with six fascial sutures. The authors reported no PSH events at a 36-month follow-up and no severe postoperative complications. However, the cohort was characterized by a notably low median BMI (22.5), with only two patients presenting a BMI > 25. This is noteworthy, as all studies evaluating non-mesh prophylactic strategies reported a mean BMI below 25, which may represent a potential source of bias given that obesity is a major risk factor for PSH development. Zhou et al. recently published the first RCT comparing EP of the ileal conduit with the conventional technique [19]: at a median follow-up of 32 months, the authors observed a significantly lower incidence of radiological PSH in favor of the modified technique (11.5% vs. 28.8%; *p* = 0.028; HR 0.374, 95%CI 0.145–0.965). Importantly, the EP procedure did not increase operative time or surgery-related complications when compared with standard conduit placement [19]. An additional non-mesh preventive surgical method was proposed by Tanaka et al. [25]. Tanaka’s surgical technique comprises three key steps: first, creation of a conduit passage with a diameter of ≤2.4 cm; second, vertical incision of the posterior rectus sheath and peritoneum approximately 2 cm lateral to the intended stoma site to form an oblique tract for conduit placement; and third, separate fixation of the anterior rectus sheath and the posterior rectus sheath with peritoneum to the ileal conduit. Retrospective analysis demonstrated a significantly lower incidence of PSH among patients treated with the modified technique (3.5%) compared with those undergoing the conventional approach (19.6%) [25].

### 4.2. Safety Profile

Preventive surgical approaches demonstrated an overall favorable safety profile in the included studies, with reported complication rates ranging from 0.0% to 27.0%. Infectious complications were the most frequently observed. All included RCTs reported a comparable number of adverse events between the experimental and control cohorts. Furthermore, only one patient across all studies experienced a severe procedure-related complication attributable to mesh placement, specifically partial ischemia of the ileal loop [13]. These findings should, however, be interpreted with caution, as evidence regarding long-term outcomes remains limited. Late urostomal complications following mesh placement are poorly documented in the literature, and no long-term safety data are currently available for non-mesh preventive techniques. In a recent retrospective study, Jakobsson et al. evaluated long-term outcomes after reparative surgery for ileal conduit PSH using mesh reinforcement [21]: thirteen of 25 patients (52%) experienced either a urostomal complication, PSH recurrence, or both. Mesh erosion occurred in four patients, three of whom were diagnosed more than five years postoperatively. Urostomal stenosis developed in four patients, and five required additional reparative surgery [21].

Severe complication rates were lower among cohorts undergoing non-mesh preventive techniques (0.0–8.8%), with no procedure-related complications reported. One case of mild stomal ischemia occurring five days postoperatively required emergent laparotomy, although this event was attributed to severe ileus rather than the surgical method itself [27]. The same authors reported two cases of urinary leakage requiring re-intervention, with no significant differences in safety outcomes among open, laparoscopic, and robot-assisted approaches [27].

From a theoretical perspective, non-mesh techniques may offer improved safety by avoiding prosthesis-related complications. However, the absence of comparative studies precludes definitive conclusions regarding their relative safety.

### 4.3. Limitations

Our review is subject to several limitations. First, substantial procedural heterogeneity was observed across the included studies. Authors described various mesh placement techniques using different mesh materials (e.g., absorbable, alloplastic) and adopted diverse surgical approaches (open vs. minimally invasive surgery). The lack of a standardized surgical pathway inevitably affects the consistency and comparability of the findings. Second, most of the included studies were retrospective in design. Of the 12 papers reviewed, only three were RCTs—two evaluating mesh-based procedures and one assessing a non-mesh technique. Moreover, a considerable proportion of studies consisted of small case series, thereby limiting the overall methodological strength of the available evidence. Third, follow-up methodologies and definitions of PSH onset were inconsistently applied across studies. Considerable variability exists in how parastomal hernia is defined in literature, with some studies relying on clinical examination, others on CT imaging, and still others using a combination of both. Importantly, CT imaging tends to overestimate parastomal hernia prevalence compared with clinical evaluation, thereby leading to artificially inflated incidence rates. Furthermore, definitions of clinical and radiological PSH recurrence varied substantially among the included studies, and the lack of standardized criteria for “PSH recurrence” may introduce significant bias in the assessment of surgical outcomes, as previously discussed. Finally, follow-up durations were generally insufficient to adequately assess long-term outcomes and late-onset complications, further limiting the strength of the conclusions that can be drawn.

## 5. Conclusions

PSH following ileal conduit urinary diversion represents a complex clinical scenario, associated with potentially severe morbidity and significant impairment in quality of life. The procedural peculiarities of the ileal conduit, together with the limited availability of robust evidence, continue to render its surgical management challenging. Findings from the included studies suggest encouraging results in terms of both recurrence rates and safety outcomes. Overall, preventive strategies have demonstrated moderate effectiveness in reducing the risk of PSH after RC, with RCTs reporting favourable outcomes for both mesh-based and non-mesh approaches compared with conventional techniques. However, the limited methodological quality of the current literature precludes the formulation of definitive clinical recommendations. Enhanced preoperative patient selection may help identify individuals at increased risk of PSH development, allowing surgical strategies to be tailored accordingly. In this context, the principles of personalized medicine may offer a valuable framework for optimizing PSH prevention strategies following ileal conduit urinary diversion. By integrating patient-specific factors—such as comorbidities, body composition, nutritional status, prior abdominal surgery, and potential biological markers of impaired wound healing—clinicians may better stratify PSH risk and individualize preventive interventions. A tailored approach could facilitate more informed decision-making regarding the use of mesh-based versus non-mesh techniques, as well as the selection of patients most likely to benefit from prophylactic measures. Patients deemed at higher risk may be counselled regarding participation in ongoing RCTs evaluating prophylactic mesh placement or EP of the ileal conduit. Nevertheless, despite early promising results, patients should be informed of the potential for long-term procedure-related complications. In this context, non-mesh preventive approaches may offer the theoretical advantage of avoiding mesh-related adverse events, such as contamination or dislocation. At present, however, available data are insufficient to support direct comparison between mesh and non-mesh prophylactic strategies. Larger studies with standardized methodologies and extended follow-up periods are required to generate high-quality evidence and clarify the optimal preventive approach for PSH in patients undergoing RC with ileal conduit urinary diversion.

## Figures and Tables

**Table 1:** Main characteristic of the included studies.

Author	Year	Design	Journal	Risk of Bias
Djaladat [18]	2024	RCT	The Journal of Urology	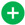
Tanaka [25]	2024	RS	International Journal of Urology	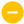
Zhou [19]	2023	RCT	Cell Reports Medicine	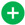
Atwater [20]	2022	RS	Urology	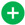
Jakobsson [21]	2022	RS	Scandinavian Journal of Urology	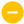
Li [26]	2022	RS	Urologic Oncology: Seminars and Original Investigations	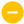
Jiang [22]	2021	PS	The Journal of Urology	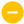
Liedberg [13]	2020	RCT	European Urology	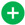
Li [27]	2019	RS	Cancer Communications	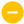
Tenzel [23]	2018	RS	Hernia	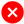
Styrke [24]	2015	PS	Scandinavian Journal of Urology	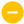
Zhang [28]	2010	RS	Urology	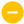

Abbreviations as follow: RS = retrospective study; PS = prospective study; RCT = randomized controlled trial. Legend: 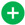 = low risk; 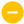 = some concerns; 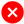 = high risk.

**Table 2:** PSH prevention with prophylactic mesh placement *.

General Information				Surgical Information							Post-Operative Information				
Author	Pat. (n.)	Age, yr, med.	BMI, kg/m^2^, med.	Surgical Approach			CDC Grade ≥ III at 90 d. n. (%)	LOS, med., d.	Mesh-Related Complications n. (%)	Follow Up, med., mo	PSH Recurrence n. (%)			Re-Intervention	
				VLS n. (%)	Open n. (%)	RA n. (%)					RR	CR	NS	n. (%)	Indication
Djaladat [18]	72	76 (71–80)	26.6 (22.9–30.6)	0 (0.0)	38 (53.0)	34 (47.0)	10 (14.0)	5 (4–8)	0 (0.0)	24.0 (17.0–26.0)	18 (25.0)	8 (11.1)	/	3 (4.2)	PSH recurrences
Atwater [20]	11	NR	NR	0 (0.0)	11 (100.0)	0 (0.0)	0 (0.0)	NR	0 (0.0)	5.4 (0.8–8.0)	NR	NR	2 (18.2)	1 (9.0)	Stomal stenosis
Jakobsson [21]	11	NR	NR	0 (0.0)	11 (100.0)	0 (0.0)	NR	NR	NR	72.0 (29.0–92.0)	NR	NR	4 (36.4)	1 (9.0)	Symptomatic PSH recurrence
Jiang [22]	38	NR	NR	0 (0.0)	4 (10.5)	34 (89.5)	0 (0.0)	NR	0 (0.0)	4.0 (2.0–8.0)	0 (0.0)	1 (2.6)	/	NR	/
Liedberg [13]	118	71 (65–74)	26.0 (22.0–28.0)	0 (0.0)	118 (100.0)	0 (0.0)	32 (27.0)	NR	1 (0.8)	36.0 (24.0–60.0)	17/92 (19.0)	10/92 (11.0)	/	3 (3.3)	Two PSH recurrences, 1 stoma local revision
Tenzel [23]	18	68 (NR)	NR	0 (0.0)	0 (0.0)	18 (100.0)	0 (0.0)	NR	0 (0.0)	11.0	0 (0.0)	0 (0.0)	/	0 (0.0)	/
Styrke [24]	114	69 (±7)	26 (±4)	0 (0.0)	114 (100.0)	0 (0.0)	0 (0.0)	NR	0 (0.0)	32.0	NR	8 (7.0)	/	0 (0.0)	/

Abbreviations as follow: Pat. = patients; n. = number; BMI = body mass index; CDC = Clavien-Dindo complications; LOS = length of stay; d. = days; mo = months; yr = years; med. = median; PSH = parastomal hernia; VLS = video-laparoscopic; RA = robot-assisted; RR = radiological recurrence; CR = clinical recurrence; NS = not specified; NR = not reported; / = not applicable. * Numbers within brackets indicate ranges, unless otherwise stated.

**Table 3:** PSH prevention without prophylactic mesh placement *.

Surgical Information											Post-Operative Information				
Author	Pat. (n.)	Age, yr, med.	BMI, kg/m^2^, med.	Procedure	Surgical Approach			CDC Grade ≥ III at 90 d. n. (%)	LOS, med., d.	Procedure-Related Complications n. (%)	Follow-Up, med., mo	PSH Recurrence, n. (%)			Re-Intervention n. (%)
					VLS n. (%)	Open n. (%)	RA n. (%)					RR	CR	NS	
Tanaka [25]	57	72 (±9)	23.1 (3.4)	** NSP	0 (0.0)	45 (78.8)	12 (21.2)	5 (8.8)	NR	0 (0.0)	22 (13–28)	2 (3.5)	NR	/	0 (0.0)
Zhou [19]	52	63 (±12)	23.0 (3.1)	EPIC	0 (0.0)	18 (34.6)	32 (61.5)	2 (3.8)	3 (5.8)	11 (±5.3)	0 (0.0)	32 (15)	6 (11.5)	7 (12.2)	NR
Li [26]	241	63 (18–88)	23.0 (3.3)	EPIC	0 (0.0)	241 (100.0)	0 (0.0)	NR	NR	0 (0.0)	25.5 (6–159)	16 (7.5)	NR	/	0 (0.0)
Li [27]	145	62 (18–89)	21.8 (3.2)	EPIC	0 (0.0)	145 (100.0)	0 (0.0)	7 (4.8)	NR	0 (0.0)	26.0 (6–156)	0 (0.0)	0 (0.0)	/	0 (0.0)
Zhang [28]	56	62 (35–81)	22.5 (NR)	EPIC	0 (0.0)	56 (100.0)	0 (0.0)	0 (0.0)	NR	0 (0.0)	36.0 (8–96)	0 (0.0)	0 (0.0)	/	0 (0.0)

Abbreviations as follow: Pat. = patients; d. = days; n. = number; CDC = Clavien-Dindo complications; mo = months; yr = years; med. = median; PSH = parastomal hernia; EPIC = extraperitonealization of the ileal conduit; NSP = novel surgical procedure for ileal stoma creation; VLS = video-laparoscopic; RA = robot-assisted; RR = radiological recurrence; CR = clinical recurrence; NS = not specified; NR = not reported; / = not applicable. * Numbers within brackets indicate ranges, unless otherwise stated. ** Key points of the novel procedure are described by the Authors as follow: (1) the passage of the ileal conduit is ≤2.4 cm in diameter in principle; (2) the posterior rectus sheath and peritoneum are vertically incised 2 cm laterally from the middle of the stoma site to make an oblique passage for the ileal conduit; and (3) the anterior rectus sheath and posterior rectus sheath with peritoneum are fixed to the ileal conduit separately.

## Data Availability

The raw data supporting the conclusions of this article will be made available by the authors on request.

## Supplementary Materials

The following supporting information can be downloaded at: https://www.mdpi.com/article/10.3390/jpm16010040/s1, File S1: PRISMA checklist [46]; File S2: Review search strategy; Table S1: Methodological quality assessment for RCTs (CASP items) [13,18,19]; Table S2: Methodological quality assessment for non-randomized studies (MINORS items) [20,21,22,23,24,25,26,27,28].

## Abbreviations

The following abbreviations are used in this manuscript:

RC	Radical Cystectomy
MIBC	Muscle-Invasive Bladder Cancer
PSH	Parastomal Hernia
PRISMA	Preferred Reporting Items for Systematic Reviews and Meta-Analyses
CT	Computed Tomography
RCT	Random Controlled Trial
CASP	Critical Appraisal Skills Programme
MINORS	Methodological Index for Non-Randomized Studies
EP	Extraperitonealization
HR	Hazard Ratio
CI	Confidence Interval
OR	Odds Ratio
