# Supplementary material for: Preventing Parastomal Hernias After Radical Cystectomy with Ileal Conduit: A Systematic Review Regarding Surgical Prophylactic Techniques
Source: J Pers Med. 2026 Jan 8;16(1):40. doi: 10.3390/jpm16010040 (PMC12842995; doi:10.3390/jpm16010040)
Supplement: Supplementary file 1 [file jpm-16-00040-s001.zip › Supplemenary material table S2 MINORS items.pdf]

Table S2. Methodological quality assessment for non-randomized studies.

	MINORS items*											
	(1) A clearly stated aim	(2) Inclusion of consecutive patients	(3) Prospective collection of data	(4) Endpoints appropriate to the aim of the study	(5) Unbiased assessment of the study endpoint	(6) Follow-up period appropriate to the aim of the study	(7) Loss to follow up less than 5 %	(8) Prospective calculation of study size	**(9) An adequate control group	**(10) Contemporary groups	**(11) Baseline equivalence of groups	**(12) Adequate statistical analyses
Atwater [20] (2022)	2	2	0	2	2	1	2	1	N/A	N/A	N/A	N/A
Jakobsson [21] (2022)	2	2	0	1	1	2	0	0	N/A	N/A	N/A	N/A
Jiang [22] (2021)	2	0	2	2	0	1	0	0	N/A	N/A	N/A	N/A
Tenzel [23] (2018)	2	0	0	2	0	1	0	0	N/A	N/A	N/A	N/A
Styrke [24] (2015)	2	2	0	2	2	2	0	1	N/A	N/A	N/A	N/A
Tanaka [25] (2024)	2	1	0	2	1	1	0	0	2	2	0	2
Li [26] (2022)	2	2	0	2	1	1	2	1	1	2	1	1
Li [27] (2019)	2	2	0	2	1	1	0	2	2	0	2	2
Zhang [28] (2010)	2	2	0	2	1	1	0	1	N/A	N/A	N/A	N/A

Abbreviations as follow: MINORS = methodological index for non-randomized studies; N/A = not applicable. *Item score: 0 (not reported), 1 (reported but inadequate), 2 (reported and adequate). Ideal total score for comparative studies should reach 16 points. ** Additional criteria in case of comparative studies.
