# Supplementary material for: Preventing Parastomal Hernias After Radical Cystectomy with Ileal Conduit: A Systematic Review Regarding Surgical Prophylactic Techniques
Source: J Pers Med. 2026 Jan 8;16(1):40. doi: 10.3390/jpm16010040 (PMC12842995; doi:10.3390/jpm16010040)
Supplement: Supplementary file 1 [file jpm-16-00040-s001.zip › Supplementary material File S2 _ search strategy.pdf]

MEDLINE/PUBMED

Search date: 30 December 2024

Search terms: ((Cystectomy OR Radical Cystectomy OR Urinary Diversion OR Ileal Conduit OR Bricker OR Urostomy OR Stoma) AND (Parastomal Hernia OR Hernia OR Hernia Prevention))

Total matches: 1526.

SCOPUS

Search date: 30 December 2024

Search terms: (cystectomy OR (ileal AND conduit) OR urostomy OR (urinary AND diversion)) AND parastomal AND hernia AND prevention)

Total matches: 287.

Cochrane Central Register of Controlled Trials (CENTRAL)

Search date: 30 December 2024

Search terms: (parastomal hernia OR radical cystectomy OR urostomy OR urinary diversion) in "All Text"

Total matches: 1865.

WEB OF SCIENCE

Search date: 30 December 2024

Search terms: ALL FIELDS: (radical cystectomy OR ileal conduit OR urostomy) AND ALL FIELDS: (hernia OR parastomal hernia)

Total matches: 304.
