# Supplementary material for: Preventing Parastomal Hernias After Radical Cystectomy with Ileal Conduit: A Systematic Review Regarding Surgical Prophylactic Techniques
Source: J Pers Med. 2026 Jan 8;16(1):40. doi: 10.3390/jpm16010040 (PMC12842995; doi:10.3390/jpm16010040)
Supplement: Supplementary file 1 [file jpm-16-00040-s001.zip › Supplementary material table S1 CASP items RCTs.pdf]

Table S1. Methodological quality assessment for RCTs.

	CASP Randomised Controlled Trial Standard Checklist items*										
	**(1) Did the study address a clearly focused research question?**	**(2) Was the assignment of participants to interventions randomised?**	**(3) Were all participants who entered the study accounted for at its conclusion?**	**(4) Were partecipants, investigators and people assessing results "blinded"?**	**(5) Were the study groups similar at the start of the randomised controlled trial?**	**(6) Apart from the experimental intervention, did each study group receive the same level of care (that is, were they treated equally)?**	**(7) Were the effects of intervention reported comprehensively?**	**(8) Was the precision of the estimate of the intervention or treatment effect reported?**	**(9) Do the benefits of the experimental intervention outweigh the harms and costs?**	**(10) Can the results be applied to your local population/in your context?**	**(11) Would the experimental intervention provide greater value to the people in your care than any of the existing interventions?**
Djadalat [18] (2024)	Y	Y	N	N	Y	Y	Y	Y	Y	Y	CT
Liedberg [13] (2020)	Y	Y	N	N	Y	Y	Y	Y	Y	Y	CT
Zhou [19] (2023)	Y	Y	Y	N	Y	Y	Y	Y	Y	Y	Y

Abbreviations as follow: CASP = Critical Apprisal Skills Programme; Y = yes; N = no; CT = can't tell. *Item score: yes; no; can't tell.
